# Planetary health and Indigenous sovereignty: exploring the theory of change of the Healthy Environments and Lives (HEAL) network in Western Australia

**DOI:** 10.1136/bmjpo-2024-002894

**Published:** 2025-05-19

**Authors:** Lucie O’Sullivan, Theoni Whyman, Mara West, Noel Nannup, Jaime Yallup Farrant, Naomi Joy Godden, Emma-Leigh Synnott, Raewyn Mutch, Anita J Campbell, Kylie Wrigley, Brad Farrant

**Affiliations:** 1 The University of Western Australia, Law School, Perth, Western Australia, Australia; 2 The Kids Reserach Institute Australia, University of Western Australia, Perth, Western Australia, Australia; 3 Univeristy of Western Australia, Centre for Child Health Research, Perth, Western Australia, Australia; 4 Climate Justice Union, Perth, Western Australia, Australia; 5 Centre for People, Place and Planet, Edith Cowan University School of Arts and Humanities, Bunbury, Western Australia, Australia; 6 Fiona Stanley and Fremantle Hospital Group, South Metropolitan Health Service, Perth, Western Australia, Australia; 7 Paediatrics, Perth Children’s Hospital, Nedlands, Western Australia, Australia; 8 Department of Infectious Diseases, Perth Children’s Hospital, Perth, Western Australia, Australia; 9 The University of Western Australia School of Medicine and Pharmacology, Perth, Western Australia, Australia

**Keywords:** Qualitative research

## Abstract

This paper outlines the theory of change which underpins the Western Australian (WA) hub of the Healthy Environments and Lives (HEAL) network. HEAL is an Australian national research initiative that aims to address the health impacts of climate and environmental change. The WA hub’s theory of change is focused on improving the health and well-being of the planet and people, including children, through centring Indigenous sovereignty, voices and ways of knowing and being in research, policy development and service provision. The WA hub also recognises it is essential for place-based, community-led solutions, which strengthens responses to climate and environmental change, grounding mitigation and adaptation efforts in local priorities, knowledges and relationships. To action its theory of change, the HEAL WA hub has embraced Community-Based Participatory Action Research (CBPAR), which positions Aboriginal elders, people with diverse lived experiences, young people, community organisations and policy makers as co-researchers. This weaving together of different ways of knowing, grounded in holistic, relational and multigenerational worldviews, enables community members to lead change and hold decision-making power at all stages of research. Through CBPAR, researchers, community members and organisations, policy-makers and service providers build and foster meaningful relationships and collaborate to co-design, implement and translate research. Young people and children are a vital part of the work, and their voices and priorities are integrated in all phases of the work to ensure intergenerational justice and vision also guides practice. This ensures HEAL WA can affect targeted, research-driven and equitable community-led change both now and for generations to come.

**WHAT IS ALREADY KNOWN ON THIS TOPIC**

- As climate change and its impacts reach crisis point, there has been a call globally to incorporate Indigenous knowledges into climate adaptation research and policy. However, there has been little attention as to how this is done in a way that ensures respectful and genuine collaboration with Indigenous people and avoids tokenism, extractive practices and one-way knowledge transfer.

**WHAT THIS STUDY ADDS**

- This paper details the theory of change behind the decolonised, Indigenous Led and Community-Based Participatory Action Research (CBPAR) process used in the Healthy Environments and Lives Western Australian research network and demonstrates how to genuinely centre the voices and knowledge of Indigenous peoples and others with diverse lived experiences, including children and young people.

**HOW THIS STUDY MIGHT AFFECT RESEARCH, PRACTICE OR POLICY**

- The genuine collaboration of researchers and policy-makers with Indigenous peoples and peoples with diverse lived experiences, including young people, through an Indigenous led CBPAR approach ensures climate research and policy development reflects the diverse needs of the peoples with whom the team collaborates. It also ensures that the work is grounded in relational, respectful and reciprocal ways of understanding. The paper articulates the theory and presents our experience and practice in this way of working, as a methodology of how respectful weaving of knowledges toward planetary health can be achieved in practice.

## Introduction

 Our shared home, Earth, is currently facing a health emergency.^1^ Anthropocentric environmental changes, including species extinction, biodiversity loss, pollution and climate change, are creating immense challenges for the health and well-being of peoples and communities globally.^2 3^ These challenges are felt most acutely by communities living with intersecting inequalities, such as Indigenous peoples (The term ‘Indigenous’ refers to Indigenous people globally, whereas terms such as ‘Aboriginal’ and ‘Māori’ are used to refer to the Indigenous people of Australia and New Zealand, respectively), who carry a disproportionate burden of mortality and morbidity emanating from colonisation and entrenched systems of dispossession and disempowerment.^1 4^

In addition, children and young people, including children yet to be born, will bear a disproportionate burden of the impacts of climate change.^5^ Children already suffer around 90% of the burden of disease associated with climate change due to smaller body size, immature neurological and physical development, emerging immune systems, and higher metabolic and respiratory rates.^5^ The mental health of children and young people is also adversely affected by climate change, with increasing temperatures and growing extreme weather events resulting in high rates of psychological distress, mood and anxiety disorders, and suicide.^6^ With the rapid escalation of the issues of climate change and concurrent deterioration of ecosystems and life-support systems, the future of livability of many locations across the planet comes into question.^7^ Clearly, this is an issue of intergenerational equity.^8^ With the health and survival of humanity, and all other life forms in jeopardy, there is a need for equitable and immediate action to mitigate and adapt to climate change.

The most recent Intergovernmental Panel on Climate Change synthesis report, published in 2023, identifies Indigenous knowledges as a ‘major resource for adapting to climate change’ and calls for the integration of such knowledges into existing and future climate mitigation and adaptation planning.^9^ While there is a global call to incorporate Indigenous knowledges into climate change and adaptation research and policy,^19^,^11^ the question of how to do this appropriately and respectfully is not so well attended to. Current research and policy development are largely underpinned by colonial mandates and grounded in anthropocentric epistemologies and ontologies, are extractive, mechanistic and linear in nature. We often see these processes giving little knowledge or information back to the peoples they consult, and there is little consideration of how the work nests within or relates to the more-than-human, or exploration of deeper two-way learning and reflection spaces.^2 12^ Alternatively, Indigenous epistemologies and ontologies are inherently holistic, collective and place-based. Thus, if we are to integrate Indigenous knowledges into research and policy, to ensure it is not tokenistic or superficially merged, the work demands a shift away from extractive processes towards one that is itself reciprocal, relational, reflective and collective.^2 12^

This respectful weaving of Indigenous and non-Indigenous ways of knowing and being requires non-Indigenous researchers to yield space, decolonise processes and cede power to Indigenous voices and practices. This includes creating an enabling research and policy environment with collaborative Indigenous and non-Indigenous ethics and governance frameworks to facilitate the redistribution of power, decision-making and resources. This also enables a shift from short-term thinking and decision making towards expanded concepts of health, and more long-term and intergenerational solutions and associated health outcomes with, and for, the community.

In response to the call to action for the respectful weaving of Indigenous and non-Indigenous knowledge systems, the Healthy Environments and Lives (HEAL) network was established in Australia in 2021. This research network, with its commitment to democratic decision-making and strong Aboriginal and Torres Strait Islander leadership,^13^ aims to strengthen the resilience of health systems to climate change-related risks, protect and improve public health, and reduce health inequities and inequalities in the context of climate change.^13^

The HEAL Network in Western Australia (WA) is based on a theory of change that through strengthening Indigenous sovereignty, we improve the health and well-being of the planet. This is done by incorporating Indigenous voices and ways of knowing in research spaces, policy development and service provision. The team’s work with local Noongar (Noongar, also spelt Nyoongar, is the Aboriginal language and cultural group of southwest WA) Elders and Aboriginal community members more broadly has led to an increased all-encompassing understanding of Indigenous sovereignty, referring to a broad ethics of care for all people and beings. Therefore, by centring Aboriginal voices and ways of knowing, it is not only Aboriginal people who benefit but all of Country, including all who live in and on Country. This includes youth and people who are often marginalised and disproportionately impacted by climate and environmental change, referred to as people with diverse Lived Experiences (The term ‘people with diverse Lived Experiences’ includes young people, disabled people, queer folk, people in poverty, and people with a refugee background with whom the team works. Our research intentionally includes people from these groups to ensure our work reflects their needs and priorities. Lived Experience is capitalised as their involvement prioritises bringing their lived and living experience and knowledges as a specific named role). Additionally, the HEAL WA work is also grounded in the understanding that adequate responses to climate and environmental change, in addition to requiring Indigenous sovereignty, must include epistemological pluralism and place-based, community-led solutions. To do this, HEAL WA has embraced Community-Based Participatory Action Research (CBPAR), enabling Aboriginal Elders, community organisations, young people and people with diverse Lived Experiences to co-design and shape research and activities of the network.

This article explores the development of this WA Network theory of change and the process of establishing practices and protocols that respectfully weave together Indigenous and western knowledges. In this, we first outline Indigenous concepts of planetary health, decolonising research, and CBPAR, before demonstrating these theories in use in the HEAL WA hub theory of change. We finish with concluding statements on how this way of working not only fulfils the aims of the wider HEAL network, but highlights our hope of sharing this methodology to assist others in the ‘how’ of respectfully weaving Indigenous and Western knowledges and the role in human health, planetary health and the health of future generations.

However, before we go on, we must take a moment to position the authors.^14^ The lead author, Lucie, is a Kāi Tahu Māori woman with a background in law and political philosophy. Theoni Whyman is a Paakantji woman, a basket weaver and has a background in psychology, Social and Emotional Well-being (SEWB), and qualitative health research. Aunty Mara West is a Yamatji Elder with extensive experience working as a cultural advisor on numerous research projects. She is passionate about changing the paradigm for sustainable water and energy systems in remote communities. Uncle Noel Nannup is a well-respected Nyoongar and Indjibarndi Elder and academic with extensive cultural wisdom and knowledge. Jaime Yallup Farrant is a Wadjella (The term Wadjella is the Noongar word for a white non-Aboriginal person. It is not capitalised as it is not a specific name, but rather a generic term that represents a wide cluster of people) woman born in England, with a background in participatory arts-based community development. Naomi Godden is a Wadjella climate justice activist and scholar who lives and works on the unceded lands of the Wardandi Noongar people. Emma-Leigh Synnott, a Wadjella woman born in Boorloo and living on unceded Whadjuk Noongar Boodja, is a medical doctor, academic, educator and community organiser working at the intersections of planetary health, ecological and social justice and human health and well-being. Raewyn Mutch is a Kāi Tahu Māori woman and paediatrician with a background in refugee health, neurodiversity and youth justice, global mental health and healing. Anita Campbell is a Wadjella child health doctor and researcher working in infection management and prevention. Kylie is a Wadjella woman, a white settler from southern Africa with a background in climate and sustainability organising, research and education. Brad Farrant is a Wadjella man and WA regional lead for the HEAL Network with a background in developmental psychology and elder-led and community-led research. All authors have involvement in the HEAL WA network.

## Indigenous Knowledges and planetary health

It is well established that Western and Aboriginal concepts of health are fundamentally different.^4^ Western conceptualisations of health focus on individual biological factors, relegating environmental, cultural, spiritual and socioeconomic considerations to subsidiary determinants. In contrast, Aboriginal concepts are more holistic and eco-centric.^4^ Commonly referred to as SEWB, health is understood in relation to country, community and environmental well-being and encompasses spiritual, cultural and emotional elements. Health is also considered in the context of intergenerational (past and future) family and community health.^4 15 16^ This understanding of health, which privileges our relations with the natural world, is echoed by many Indigenous communities globally, including the Māori people of Aotearoa/New Zealand^17^ and the First Nations people of Canada.^18^ Indigenous peoples understand health to be intimately interconnected to value systems, community well-being and the natural world.^18^ These notions inform the ways respective cultures conceptualise planetary health.

From a Western perspective, planetary health is defined as ‘the health of human civilisation and the state of the natural systems on which it depends’.^19^ This framing is influenced by highly anthropocentric epistemologies and ontologies; wherein human health tends to be viewed as separate from, and superior to, the health of broader ecosystems and the natural world. This centring and privileging of humans over the rest of the natural world speaks to the colonial, extractive and Western systems and worldviews that created anthropogenic climate change.^19^ Planetary health from an Indigenous perspective radically departs from the hierarchy of beings present in the Western paradigm. From an Indigenous perspective, there are deep, reciprocal relations between humans and the land, waters, animals and surrounding eco-systems. Rather than using and exploiting the planet for their own benefit, humans live with, and care for, the planet, inclusive of more-than-human ecosystems and kin. Human health and well-being are therefore not separate from the health of the planet but are fundamentally interconnected to it, meaning all aspects of the natural world are equally privileged and valued.^2 19 20^ In addition, Indigenous concepts of planetary health have an intergenerational focus, recognising the need to safeguard the health and well-being of all beings present now and those not yet born, including children. This ensures they will thrive and inherit a safe and healthy planet to call home. In embracing this eco-centric, relational conceptualisation of planetary health, there is the opportunity to more meaningfully align human, cultural, social and environmental well-being across time and generations.^21 22^

**Figure 1:**
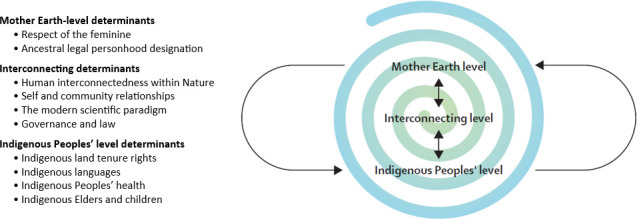
The determinants of planetary health and how they interconnect. Used with permission.^2^

Building on this, although determinants of physical health are well-known within a Western public health discourse, there is a paucity of discussion regarding determinants of planetary health, especially those grounded in Indigenous knowledge systems.^2^ In a paper titled *The Determinants of Planetary Health: An Indigenous Consensus Perspective,* Indigenous scholars, practitioners, elders and knowledge-holders from multiple nations came together to define the determinants of planetary health from an Indigenous perspective. The 10 determinants (see figure 1) represent interconnected ‘factors and conditions that affect the health of the planet’ from an eco-centric worldview.^2^ The determinants speak to the importance of combating existing colonial mandates and systems of oppression, recognising the interdependence between humans and the natural world, adopting Indigenous-led governance models, and upholding First Law (First Law refers to the laws of the land as laid out by the creator, as opposed to the laws of man).^2^ In outlining the determinants, the paper foregrounds the role of Indigenous peoples and knowledge systems in protecting Mother Earth and addressing climate change.

## The HEAL WA theory of change: Indigenous sovereignty and decolonisation

An Indigenous model for conceptualising planetary health and determinants of planetary health coupled with the call for the integration of Indigenous knowledges into climate change and adaptation research and policy^2 10 23^ have been pivotal in framing the HEAL WA theory of change. The theory of change is centred on Indigenous sovereignty and privileging Indigenous voices and ways of knowing in research spaces, policy development and service provision. It also focuses on the priorities of people with diverse lived experiences, including children. Effectively engaging and protecting Indigenous peoples, youth and peoples with diverse lived experiences can be done through ‘drawing on bottom-up approaches and using Indigenous knowledge(s)’.^10^ However, for the meaningful and respectful collaboration and leadership of Indigenous peoples and peoples with diverse lived experiences, systemic change grounded in Indigenous sovereignty and decolonisation is needed.

## Decolonising research

Decolonising research does not seek to fully discard the Western knowledge system. Rather, it aims to critically evaluate its paradigm and practices so as to allow for the dismantling of harmful colonial constructs and to work collaboratively to re-establish pillars that strengthen Indigenous sovereignty.^12 24^ According to Ngāti Awa and Ngāti Porou scholar, Professor Linda Tuhiwai Smith, ‘decolonisation is a process that critically engages, at all levels, imperialism, colonialism, and postcoloniality’.^24^ Decolonising research requires non-Indigenous researchers to step back from the ‘Western cultural conceptual rubric’ and place Indigenous values, attitudes, practices and ways of knowing at the centre of the research process. Professor Anne Poelina, a Nyikina Warrwa leader and scholar, similarly notes that the process and outcome of decolonising ‘requires us to explore and analyse within an Indigenous context and importantly to honour First Law’.^25^ Such exploration and analysis cannot be done without first centring Indigenous voices, experiences and leadership. As Indigenous methodologies tend to view cultural protocols and values as an ‘integral part of (the) methodology’, decolonised research approaches should create space for holistic ways of knowing and being.^24^ Caxaj acknowledges the need for a holistic approach, arguing that a crucial component for conducting meaningful research with Indigenous communities involves upholding the protocols and practices for mutuality, power-sharing and reciprocity.^26^ These protocols and practices closely align to core structures of Indigenous ontologies and represent ways to strengthen Indigenous sovereignty in research.^25 26^ Decolonising and Indigenising research spaces not only serves to respectfully integrate Indigenous knowledge into climate change and adaptation research processes. It also has the potential to assist in creating narratives of cultural resilience, power and practice.^27^

Decolonisation of research processes includes a ‘decolonising-of-the-self’. For non-Indigenous researchers, this is an important prerequisite to addressing unconscious bias and appropriately working alongside Indigenous peoples.^12 15^ Decolonising-of-the-self involves undertaking a process of self-analysis, acknowledgement and ownership of power and privilege.^11^ It requires non-Indigenous researchers to acknowledge their embodiment of Western paradigms and reflect on how their implicit assumptions and western ethnocentric views may perpetuate colonial ideologies and institutions.^12 15^ As Krusz *et al* reflect, this process encourages non-Indigenous researchers to practice cultural humility and to question and unlearn Western constructs, rather than aspire to cultural competency.^12^ With cultural humility, one acknowledges that simply knowing ‘facts’ about a culture does not permit non-Indigenous researchers to embody the worldviews and ways of knowing and being of Indigenous peoples.^12 28^ Although the self-reflection required for decolonising-of-the-self may incite feelings of discomfort, guilt and uncertainty, this vulnerability can be transformative.^12 28^ Not only does it sow seeds for dismantling colonial constructs but also allows for humanness and deeper connections to form between Indigenous and non-Indigenous researchers and communities.^12^

True decolonising-of-the-self and of research allows for a reframing of research, allows researchers to work with communities, not on communities and to share control and decision-making power with whom they work. It allows for the creation of culturally safe spaces, appropriate conditions for collaboration with Indigenous peoples, and the respectful integration of Indigenous knowledges, all essential in achieving planetary health and well-being.

## CBPAR and communities of practice

According to Caxaj, Indigenous approaches should ‘not merely [be] a gesture but instead a reframing and reorienting of the research itself toward Indigenous control, ownership and self-definition’.^26^ CBPAR lends itself well to being grounded in Indigenous voices and ways of knowing and being.^2628^,^30^ In HEAL WA, it is the Aboriginal community members and those with diverse lived experiences who serve as co-researchers and co-create our research priorities and projects. Control over the research process is shared among all researchers. The majority of the project team that is responsible for implementing the HEAL WA network is Aboriginal. Therefore, a genuine reframing and re-orienting of the research is possible, while also contributing to cultural safety within the broader group of co-researchers. While this process is intrinsically transformational, the empowerment of people with intersecting systemic injustices and experiences of marginalisation increases the potential for sustainable impacts and social change.^28 31 32^ Co-produced research with communities, including children and youth, also builds local capacity and addresses existing barriers, including systems of dispossession and disempowerment, and societal exclusion.^2 12 28^ In this way, CBPAR projects offer opportunities to bring about targeted, research-driven and equitable social change.^28 32^

## Implementation: The HEAL WA hub

Aboriginal voices and ways of knowing and being are centred in HEAL WA climate change and health research processes through cultural governance. Cultural governance refers to the power and authority of Aboriginal peoples and communities to direct and inform all decision-making processes.^33^ It extends beyond cultural advice to knowledge-based leadership in the design, delivery and monitoring of HEAL WA projects.^33^ Appropriate authority and accountability are realised through incorporating Aboriginal ways of knowing and being into all governing policies and processes in the network, enhancing the self-determination of Aboriginal peoples and amplifying their capacities for broader influence and leadership.^25 29 33^

**Figure 2:**
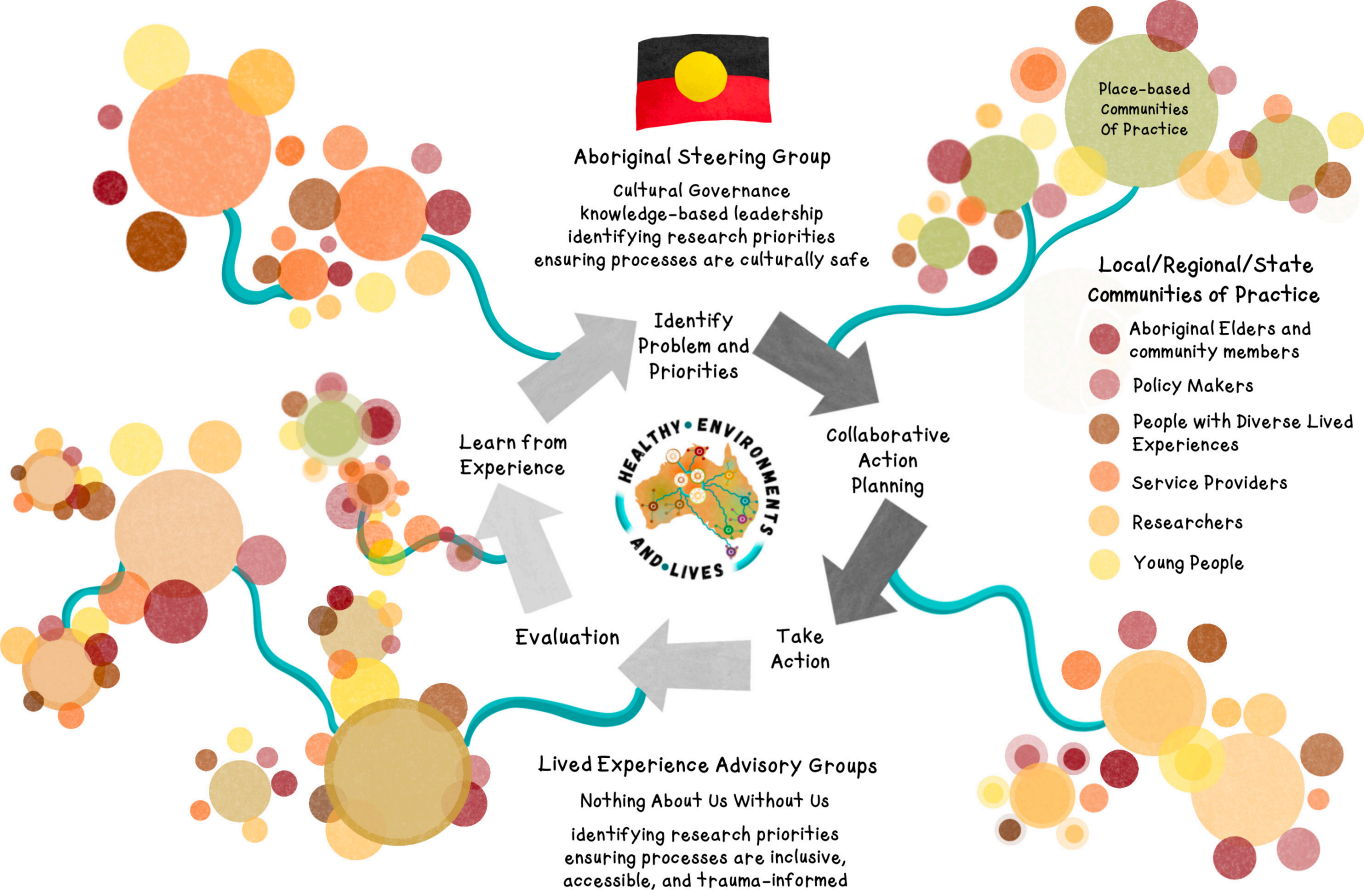
A visualisation of the HEAL WA hub’s CBPAR model. Image illustrated by Sage Ashbourne. Copyright of Climate Justice Union and HEAL WA. Used with Permission. CBPAR, Community-Based Participatory Action Research; HEAL, Healthy Environment and Lives; WA, Western Australian.

HEAL WA is composed of multiple groups (see Figure 2). The Aboriginal Steering Group (ASG) provides cultural governance and direction to the network and its projects. ASG members are Aboriginal elders, leaders, community members and researchers from across WA, including from Perth (Boorloo), Broome (Rubibi), Bunbury (Goomburrup), Albany (Kinjarling) and Collie (Koolinup). Through the ASG, children and young people were identified as a priority group as they hold disproportionate risk from climate impacts and are often ignored in governance and processes of research, policy and service provision involvement. In response to this, HEAL WA has included a Youth subgroup to their work to ensure their specific needs are addressed at all levels. In addition to the ASG, there are place-based communities of practice (CoPs) including one state-level, several regional-level and multiple local-level CoPs (each of which is convened seasonally, to align with the local Aboriginal seasons). These CoPs all attend to the need to bring local youth voices into conversations, as a priority focus. At the time of writing, these are at various stages of being formed. Place-based CoPs enable relationship building, local-led knowledges, and community-specific actions across such a large state (For context, Western Australia is approximately one-third of the size of Australia and covers an area of more than 2.5 million km^2^, about the size of western Europe. There are large distances between towns and communities). The ASG works collaboratively and in partnership with the CoPs to understand community priorities for research, translation and implementation. This structure gives control to local and regional groups and protects their right to self-determination within the HEAL WA network. The CoPs are underpinned by a strong foundation of country, community and place.^13^

The CoPs are designed to encourage a high degree of participation from diverse community members in the planning, development and implementation of research projects. The intention is for all CoPs to have the leadership and participation of Aboriginal elders, community members, representatives from government and emergency services, youth and people with diverse lived experiences, as well as academic researchers. The state CoP is assigned the specific role of supporting and empowering the local and regional CoPs. This role may be actioned through the provision of additional resources for local and regional CoPs and research projects or the identification and elimination of structural barriers to the translation and implementation of research findings. The state CoP also actively fosters relationships with local and regional CoPs to ensure trust and transparency in communication across the network. Given the key role of community members in CBPAR, it is important to adequately manage power dynamics and the diverse interests within and between the CoPs.^34^

The ASG also collaborates directly with external stakeholder groups, such as state-level and national-level government departments, policy-makers and at times industry representatives, corporate bodies and media. They may be engaged for co-design, agenda sharing, discussion and resource acquisition. The widespread influence and impact of these stakeholders can be leveraged by HEAL WA to support the translation and implementation of research findings and realise positive, transformative change. However, navigating complex power dynamics between stakeholders may be challenging. Managing stakeholder engagement with CoPs is essential to ensure equitable collaboration between members and the vital roles of country, communities and place are upheld.

## Conclusions

The respectful and genuine integration of Indigenous knowledges into climate change mitigation and adaptation research, policy development and service provision is essential for the future health of all peoples and the planet. Having the voice of children and youth heard is also considered essential. However, this has seldom been achieved in climate change mitigation and adaptation research and policy, and there is a paucity of detail on the methodology or process of this work. The HEAL WA network, however, has established this process. In recognising the importance of centring Indigenous voices and ways of knowing, Aboriginal sovereignty and epistemological pluralism, HEAL WA has embraced CBPAR as best practice within the network and projects. The HEAL WA network facilitates the respectful weaving and co-production of knowledge between Indigenous and non-Indigenous researchers and policy actors. It does this by positioning Aboriginal elders, community members, youth and local organisations as co-researchers. Through knowledge exchange and collaborative decision-making between the HEAL WA ASG, the youth subgroup and the CoPs, the WA hub seeks to support place-based, community-led solutions to the health impacts of climate and environmental change. The theory of change described here aims to ensure research projects are designed and implemented in accordance with local priorities, knowledges and reciprocal relationships, attend to intergenerational equity and need and are grounded in an eco-centric worldview that privileges a decolonised version of planetary health. We offer this theory of change in the hope it also serves as a demonstration and foundation for others who wish to pursue genuine collaboration with Indigenous peoples and weave Indigenous and diverse knowledges in climate change mitigation and adaptation research.

## Data Availability

No data are available.
