# Supplementary material for: Planetary health and Indigenous sovereignty: exploring the theory of change of the Healthy Environments and Lives (HEAL) network in Western Australia
Source: BMJ Paediatr Open. 2025 May 19;9(1):e002894. doi: 10.1136/bmjpo-2024-002894 (PMC12090852; doi:10.1136/bmjpo-2024-002894)
Supplement: Supplementary file 1 [file bmjpo-2024-002894.reviewer_comments.pdf]

PEER REVIEW HISTORY

BMJ Paediatrics Open publishes all reviews undertaken for accepted manuscripts. Reviewers are asked to complete a checklist review form and are provided with free text boxes to elaborate on their assessment. These free text comments are reproduced below.

ARTICLE DETAILS

**TITLE (PROVISIONAL)**	Planetary Health and Indigenous Sovereignty: Exploring the Theory of Change of the Healthy Environment And Lives (HEAL) Network in Western Australia
**AUTHORS**	O'Sullivan, Lucie; Whyman, Theoni; West, Mara; Nannup, Noel; Farrant, Jaime; Godden, Naomi; Synnott, Emma-Leigh; Mutch, Raewyn; Campbell, Anita J.; Wrigley, Kylie; Farrant, Brad

VERSION 1 - REVIEW

**REVIEWER NAME**	***Jeffrey Goldhagen***
**REVIEWER AFFILIATION**	University of Florida, Jacksonville, Pediatrics
**REVIEWER CONFLICT OF INTEREST**	N/A
**AI DISCLOSURE**	No
**DATE REVIEW RETURNED**	05-Oct-2024

**GENERAL COMMENTS**	This is an important introduction to the intersection of Indigenous culture and the response to climate change. The authors provide an excellent overview of the different perspectives that Indigenous communities bring to mitigation and adaptation strategies and the need to engage them as leaders in the global response to CC. The manuscript is quite deserving of publication with the following revisions.<br><br>Page 4/25. Line 11. Replace “which” with “that<br>Line 15. Replace “Centring” with “Centering”<br>Line 23. Comma after “relationships”<br>Page 6/25. Line 6. Remove the numbered paragraphs for the entire paper. Remove the word “the.”<br>Line 35. Add the words ….”mitigation and” in front of the word “adaptation”.<br>Line 37. Reword, “While there is a global call to…………..”<br>Line 42. Reword, “Current research and policy……..”<br>Line 46. Readers will not understand what you mean by “linear, anthropocentric paradigms. Do you need to include it. If so, perhaps you can explain the term.<br>Page 7/25. Line 3. Eliminate “although different. Different from what?<br>Line 6. Comma after “placed-based.”<br>Line 10. No coma after “being”<br>Line 24. Should “community” be capitalized<br>Line 32. Replace “national” with “Australian.”<br>Page 8/25. Line 6. What term are you referring to<br>Line15. Remove the word “here.”<br>Line 34. Assume knowledges is the plural form of Knowledge that you want to<br>use throughout the paper<br>Line 52. Remove “the”

	Page 9/25. Lines 10-38. Please determine which words/names need to be capitalized and be<br>consistent throughout.<br>Line 53. Which words need to be capitalized.<br>Page 10/25. Lines 20-27. Not sure I fully agree. Would soften the statement by replacing “underpinned” with “influenced and on line 25, the word “is” with “tends to be.”<br>Line 43. Comma after “planet,”<br>Page 11/25. Line 8. Remove “a group of”<br>Page 12/25. Should “Lived Experiences” be capitalized<br>Line 33. This is awkward, are there 3 peoples’ names<br>Line 44. There should be a period after Dr.<br>Page 13/25. Line 48. What does “reflexivity” mean<br>Page 14/25. Line 3. Replace “allows for” with facilitates<br>Line 23. Rplace, “lends itself well to be, “ with “is”<br>Line 28. Could read, “…..Lived Experiences who serve as co-researchers and who….”<br>Line 46. Should read, “…capacity to address………”<br>Page 17/25. Lines 21 and 25. add the word “mitigation” to read “….mitigation and adaptation…”<br>Line 53. Change “which” to “that.”

VERSION 1 – AUTHOR RESPONSE

Reviewer: 1
Dr. Jeffrey Goldhagen, University of Florida, Jacksonville
Comments to the Author
This is an important introduction to the intersection of Indigenous culture and the response to climate change. The authors provide an excellent overview of the different perspectives that Indigenous communities bring to mitigation and adaptation strategies and the need to engage them as leaders in the global response to CC. The manuscript is quite deserving of publication with the following revisions.

Page 4/25. Line 11. Replace “which” with “that. This has been replaced.
Line 15. Replace “Centring” with “Centering”. This has been replaced.
Line 23. Comma after “relationships”. A comma has been added.
Page 6/25. Line 6. Remove the numbered paragraphs for the entire paper. Remove the word “the.” Numbering and the word ‘the’ has been removed.
Line 35. Add the words ….”mitigation and” in front of the word “adaptation”. This has been added.
Line 37. Reword, “While there is a global call to………….” this has been reworded.
Line 42. Reword, “Current research and policy……..” this has been reworded.
Line 46. Readers will not understand what you mean by “linear, anthropocentric paradigms. Do you need to include it. If so, perhaps you can explain the term. To simplify, we’ve changed this to “anthropocentric epistemologies and ontologies”.
Page 7/25. Line 3. Eliminate “although different. Different from what? We have removed this.
Line 6. Comma after “placed-based.” A comma has been added.
Line 10. No coma after “being”. The comma has been removed.
Line 24. Should “community” be capitalized. The have added ‘the’ before community.
Line 32. Replace “national” with “Australian.” This has been replaced.
Page 8/25. Line 6. What term are you referring to. We have changed the sentence to make it clearer.
Line15. Remove the word “here.” This has been removed.
Line 34. Assume knowledges is the plural form of Knowledge that you want to
use throughout the paper

Yes, we use the term ‘knowledges’ here to emphasis that there is no one Indigenous knowledge. Indigenous peoples have their own knowledges which is as diverse as there are Indigenous peoples.
Line 52. Remove “the”. This has been removed.
Page 9/25. Lines 10-38. Please determine which words/names need to be capitalized and be consistent throughout. & Line 53. Which words need to be capitalized.

These sections have been reworked.
Page 10/25. Lines 20-27. Not sure I fully agree. Would soften the statement by replacing “underpinned” with “influenced and on line 25, the word “is” with “tends to be.” This has been amended.
Line 43. Comma after “planet,” A comma has been added.
Page 11/25. Line 8. Remove “a group of” This has been removed.
Page 12/25. Should “Lived Experiences” be capitalized. We capitalise the term ‘Lived Experience’ as the Lived Experience advocates involved in our research prefer the term to be capitalised.
Line 33. This is awkward, are there 3 peoples’ names

This sentence is referring to 1 person. We have listed her tribal affiliation before her name. We have changed this to read, “According to Ngāti Awa and Ngāti Porou scholar, Professor Linda Tuhiwai Smith”. We hope this makes it clearer.
Line 44. There should be a period after Dr. This has been added.
Page 13/25. Line 48. What does “reflexivity” mean

We have changed this to ‘self-reflection’
Page 14/25. Line 3. Replace “allows for” with facilitates This has been changed.
Line 23. Rplace, “lends itself well to be, “ with “is”

CBPAR is not specifically an Indigenous methodology and can therefore be grounded in other paradigms. We have left the sentence as it reads to reflect this.
Line 28. Could read, “…..Lived Experiences who serve as co-researchers and who….” This has been changed.
Line 46. Should read, “…capacity to address………” We have left this sentence as “capacity and address” as changing it would change the meaning of the sentence.
Page 17/25. Lines 21 and 25. add the word “mitigation” to read “…. mitigation and adaptation…” This has been changed.
Line 53. Change “which” to “that.” This has been changed
